# Genomic exploration of the fermented meat isolate *Staphylococcus shinii* IMDO-S216 with a focus on competitiveness-enhancing secondary metabolites

**DOI:** 10.1186/s12864-024-10490-0

**Published:** 2024-06-07

**Authors:** Ana Sosa-Fajardo, Cristian Díaz-Muñoz, David Van der Veken, Inés Pradal, Marko Verce, Stefan Weckx, Frédéric Leroy

**Affiliations:** Research Group of Industrial Microbiology and Food Biotechnology (IMDO), Faculty of Sciences and Bioengineering Sciences, Vrije Universiteit Brussel, Brussels, Belgium

**Keywords:** *Staphylococcus shinii*, Bacteriocin, Lactococcin 972, Competitiveness factors, Genomics, Meat fermentation, Starter culture, Genome annotation

## Abstract

**Background:**

*Staphylococcus shinii* appears as an umbrella species encompassing several strains of *Staphylococcus pseudoxylosus* and *Staphylococcus xylosus*. Given its phylogenetic closeness to *S. xylosus*, *S. shinii* can be found in similar ecological niches, including the microbiota of fermented meats where the species may contribute to colour and flavour development. In addition to these conventional functionalities, a biopreservation potential based on the production of antagonistic compounds may be available. Such potential, however, remains largely unexplored in contrast to the large body of research that is available on the biopreservative properties of lactic acid bacteria. The present study outlines the exploration of the genetic basis of competitiveness and antimicrobial activity of a fermented meat isolate, *S. shinii* IMDO-S216. To this end, its genome was sequenced, *de novo* assembled, and annotated.

**Results:**

The genome contained a single circular chromosome and eight plasmid replicons. Focus of the genomic exploration was on secondary metabolite biosynthetic gene clusters coding for ribosomally synthesized and posttranslationally modified peptides. One complete cluster was coding for a bacteriocin, namely lactococcin 972; the genes coding for the pre-bacteriocin, the ATP-binding cassette transporter, and the immunity protein were also identified. Five other complete clusters were identified, possibly functioning as competitiveness factors. These clusters were found to be involved in various responses such as membrane fluidity, iron intake from the medium, a quorum sensing system, and decreased sensitivity to antimicrobial peptides and competing microorganisms. The presence of these clusters was equally studied among a selection of multiple *Staphylococcus* species to assess their prevalence in closely-related organisms.

**Conclusions:**

Such factors possibly translate in an improved adaptation and competitiveness of *S. shinii* IMDO-S216 which are, in turn, likely to improve its fitness in a fermented meat matrix.

**Supplementary Information:**

The online version contains supplementary material available at 10.1186/s12864-024-10490-0.

## Background

*Staphylococcus shinii* has recently been described and validated as a novel species [1, 2]. The National Center for Biotechnology Information (NCBI) has meanwhile listed 16 genomes belonging to this species (Genome database; accessed May 2024), including that of the reference strain, *S. shinii* K22-5 M. Ten of these strains were reclassified from other staphylococcal species into *S. shinii*, including *S. pseudoxylosus* 14AME19 isolated from Korean fermented bean paste [3], *S. xylosus* INIFAP 005–08 and INIFAP 004–15, both isolated from cattle parasites [4], *S. xylosus* CHJ_154 isolated from raw cow’s milk, *S. xylosus* LSR_02N isolated from river water, and *S. xylosus* SNUC 416, SNUC 4611, SNUC 4554, SNUC 3812, and SNUC 4555 from a bovine mastitis collection [5]. Five other strains obtained from Korean fermented bean paste and human nasal swabs were annotated directly as *S. shinii* (BG1-2, BG2-4, KG4-6, KG4-7, and NNS4w).

*Staphylococcus shinii* thus bears a close genetic resemblance to both *S. xylosus* and *S. pseudoxylosus*, the latter also being a relatively novel species isolated first from bovine mastitis samples [6]. Both the taxonomic closeness and the sharing of similar ecological niches imply that the metabolic and functional information available for *S. xylosus* can serve as a benchmark to explore the relevance of this new and yet poorly described *S. shinii* species for food technology purposes. *Staphylococcus xylosus* belongs to the coagulase-negative staphylococci (CNS), situated within the group of Gram-positive, catalase-positive cocci (GCC) [7]. Ever since its first isolation from human skin [8], this ubiquitous species has been recovered from a wide range of environments. Although the species is commonly found among the normal mammalian skin microbiota [9], it can also act as an opportunistic pathogen leading to skin pathologies in both animals [10] and humans [11]. As such, it has been isolated from human clinical material and hospital environments [12]. Due to its association with mammalian skin, *S. xylosus* is a common member of the natural microbiota of meat and various products derived thereof. This is especially the case for cured meats, such as fermented meat products, where it is selected for because of its high salt tolerance [13–15].

In fermented meat products, *S. xylosus* can either arise spontaneously as a member of the natural meat microbiota, favoured by the fermentation conditions, or can deliberately be added as a starter culture, usually in combination with an acidifying lactic acid bacterium [16, 17]. The practice of using *S. xylosus* as a starter culture is based on the microorganism’s ability to contribute to the fermented meat colour and flavour, its capacity to limit oxidation of free fatty acids and avoid rancidity, and its adaptation to the fermented meat matrix and the stress conditions imposed thereon during processing [9, 18]. In addition to these conventional functionalities, there seems to be a potential for some strains within the GCC group to contribute to food safety based on the production of antagonistic compounds, i.e., microbial products that limit or inhibit bacterial growth of competing species or strains [19–21].

In the past, strategies that looked into starter culture use for biopreservation have mostly focused on lactic acid bacteria [22, 23], whereas information about the production of antagonistic compounds by GCC strains is scarce [24]. A screening of more than 300 GCC strains indicated that such activity is likely uncommon among GCC, as only a small minority of positive strains was found [20]. One of these few positive strains, *S. shinii* IMDO-S216, formerly classified as *S. xylosus* IMDO-S216, displayed antibacterial activity against a range of other GCC when studied through a deferred antagonism test. Another study has described the partial purification of a bacteriocin-like peptide produced by a *S. xylosus* strain and tested its inhibitory activity against other bacteria as well as its resistance to temperature and enzymatic digestion [25]. These findings, although interesting, remain of a preliminary nature in the absence of further characterization and identification. Therefore, further phenotypic and genomic explorations are needed to shed light on the identity and regulation of such antagonistic compounds within *S. shinii* metabolism. To the best of our knowledge, such research is currently not available for this species, partly due to its novelty. In the past, a genome analysis has been performed to elucidate the adaptation mechanisms that create fitness for *S. xylosus* within a meat matrix, linking it to the use of energy sources and other biological responses, but without reporting on the presence of antagonistic compounds [9]. Other characteristics of *S. xylosus* that have been elucidated through genome analysis include the presence of adhesion and biofilm related genes [26] and an increased oxidative stress resistance [9]. These abilities enhance competitiveness and offer an advantage for starter cultures that may contribute to overruling the autochthonous microbiota in a fermentative environment, and could be potentially shared by the new *S. shinii* species.

Competitiveness factors, among which antimicrobial activities against competitors can be counted [27], can be defined as the dynamic mechanisms that help bacteria respond to the challenges and fluctuations of the environment that affect their fitness [28]. For *S. xylosus*, inhibitory activity against strains of *Listeria* and *Pseudomonas*, as well as the release of an uncharacterized molecule that can inhibit the formation of *Staphylococcus aureus* biofilms, have been shown as promising examples of competitiveness factors with bioprotective impact [29, 30]. However, a more in-depth phenotypical and genomic exploration of the new species is needed to be able to fully benefit from this untapped potential.

Therefore, the aim of this study was to get a deeper insight into the antagonistic compounds and competitiveness factors available within the genome of *S. shinii* IMDO-S216, as to better understand how this strain functions within fermented meat ecosystems.

## Methods

### Bacterial strain, media, and inoculum build-up

The *S. shinii* IMDO-S216 strain used in this study, formerly classified as *S. xylosus* IMDO-S216, originated from the culture collection of the Research Group of Industrial Microbiology and Food Biotechnology (Vrije Universiteit Brussel, Brussels, Belgium). The strain was stored at -80 °C in glycerol-containing (25% v/v) brain heart infusion (BHI) medium (Oxoid, Basingstoke, Hampshire, United Kingdom). The inoculum build-up was performed as previously described [31]. Briefly, the strain was propagated twice in 10 mL of BHI media and incubated at 30 °C for 12 h. The precultures were then transferred (1% v/v) into 10 mL of BHI for a final incubation at 30 °C for 12 h.

### DNA extraction for Illumina and ONT library preparation

DNA extraction was performed as previously detailed, with some adaptations [32]. Different volumes of culture (3, 5, 10, and 25 mL) were tested to assess the optimal starting volume. A bacterial cell pellet was obtained from the centrifugation of 25 mL of BHI culture at 5,000 x *g* for 10 min at 4 °C. Genomic DNA was extracted from this pellet using the Genomic-tip 20/G kit (Qiagen, Hilden, Germany) with minor modifications. The bacterial pellet was resuspended in 1 mL of buffer B1 [with 2 µL of Rnase A added (100 mg/mL)] to which 40 µL of both a lysozyme solution (100 mg/mL; Merck, Darmstadt, Germany) and a mutanolysin solution (12.5 kU/mL; MilliporeSigma, Burlington, Massachusetts, United States of America), and 250 µL of a Qiagen proteinase K stock solution were added, followed by an incubation of 45 min at 37 °C. Deproteinization was achieved by adding 350 mL of buffer B2 to the lysate with a second incubation period of 30 min at 50 °C. The Genomic-tip 20/G column was equilibrated with 1 mL of buffer QBT and allowed to empty by gravitational flow. The prepared sample was then vortexed for 10 s at maximum speed and applied to the equilibrated column, where a combination of gravitational flow and gentle positive pressure using a disposable syringe was applied to assist the flow. The process was followed by threefold washing of the column with 1 mL of buffer QC each to remove as many contaminants as possible. The genomic DNA was eluted in 2 mL of buffer QF and precipitated with 1.4 mL of isopropanol. The precipitated DNA was recovered by inverting the tube and picked using a pipette tip, and then transferred to a tube containing 100 µL of nuclease-free water. The concentration of the extracted genomic DNA was measured with a Qubit fluorometer using the double-stranded DNA (dsDNA) high-sensitivity (HS) assay kit (Thermo Fisher Scientific, Waltham, Massachusetts, United States of America). The DNA purity was assessed with a NanoDrop ND-2000 spectrophotometer (Thermo Fisher Scientific). Based on the concentrations and the ratio of absorbance at 260/280 nm obtained, the optimal culture volume was selected.

### DNA sequencing

A combination of long-read and short-read sequencing of the genomic DNA was performed as previously detailed, with some adaptations [32]. Long reads were generated using the Oxford Nanopore Technologies’ (ONT) MinION sequencing device (Oxford Nanopore Technologies, Oxford, United Kingdom) [33]. Two µg of high-molecular-mass DNA were used as input for the ONT library preparation using the ONT ligation sequencing kit (SQK-LSK109; Oxford Nanopore Technologies), according to the manufacturer’s instructions. After loading the final library into an R9.4.1 flow cell, the sequencing run was performed on a MinION MK1b device using the MinKNOW software for data acquisition. Basecalling and barcode separation were performed with Guppy (v4.4.2) using the configuration file dna_r9.4.1_450bps_flipflop.cfg in high-accuracy GPU-accelerated mode (Oxford Nanopore Technologies). Quality check and trimming were performed using the NanoPack package (v1.1.0) [34]. First, NanoQC and NanoPlot were used for quality checking, whereupon NanoFilt was used to trim non-representative read ends and filter out low-quality reads, by applying the following parameters: q, 12 (filtering out reads with a Q-score lower than 12); headcrop, 80; and tailcrop, 60 (the two latter parameters allow trimming a certain number of nucleotides from the beginning and end of each read to avoid a biased content of nucleotide distribution and lower sequencing quality). Short-read sequencing was performed on an Illumina NovaSeq sequencing system (250 bp paired-end) (Illumina, San Diego, CA, United States of America) by the VUB/ULB BRIGHTcore sequencing core facility (Jette, Belgium). Quality check and trimming were performed with the tools FastQC (v0.11.3) [35] and Trimmomatic (v0.36) [36], with the following parameters: headcrop, 10 (trimming of 10 nucleotides from the beginning of each read); leading, 30 (trimmed bases off at the start of a read if below the specified threshold quality); trailing, 30 (trimmed bases off at the end of a read if below the specified threshold quality); slidingwindow, 4:15 (performed a sliding-window trimming, cutting off the read if the average quality of the bases in the window fell below the threshold specified); and minlen, 20 (reads under the specified length were not considered).

### Whole-genome *de novo* assembly

The quality-assessed and trimmed long- and short-reads were used to generate a hybrid assembly by using Unicycler (v0.5.0) [37]. The assembler was used in three different modes – Conservative, Normal, and Bold – and the results were visualized using Bandage [38] to compare the architecture of the assemblies, to check circularity/linearity, and to give an estimation of copy numbers of the plasmids. A pairwise comparison of the contigs generated with each of the three modes was performed using the basic local alignment search tool for nucleotides (blastn) [39, 40] to identify which contigs had been resolved due to more constrictive modes. The Public Database for Molecular Typing and Microbial Genome Diversity (PubMLST) was used to elucidate the types of replicons of the resolved plasmids by performing a plasmid multilocus sequence typing (pMLST; accessed May 2024) [41].

### Genome analysis of biosynthetic gene clusters related to antagonistic compounds and competitiveness factors

**Fig. 1:**
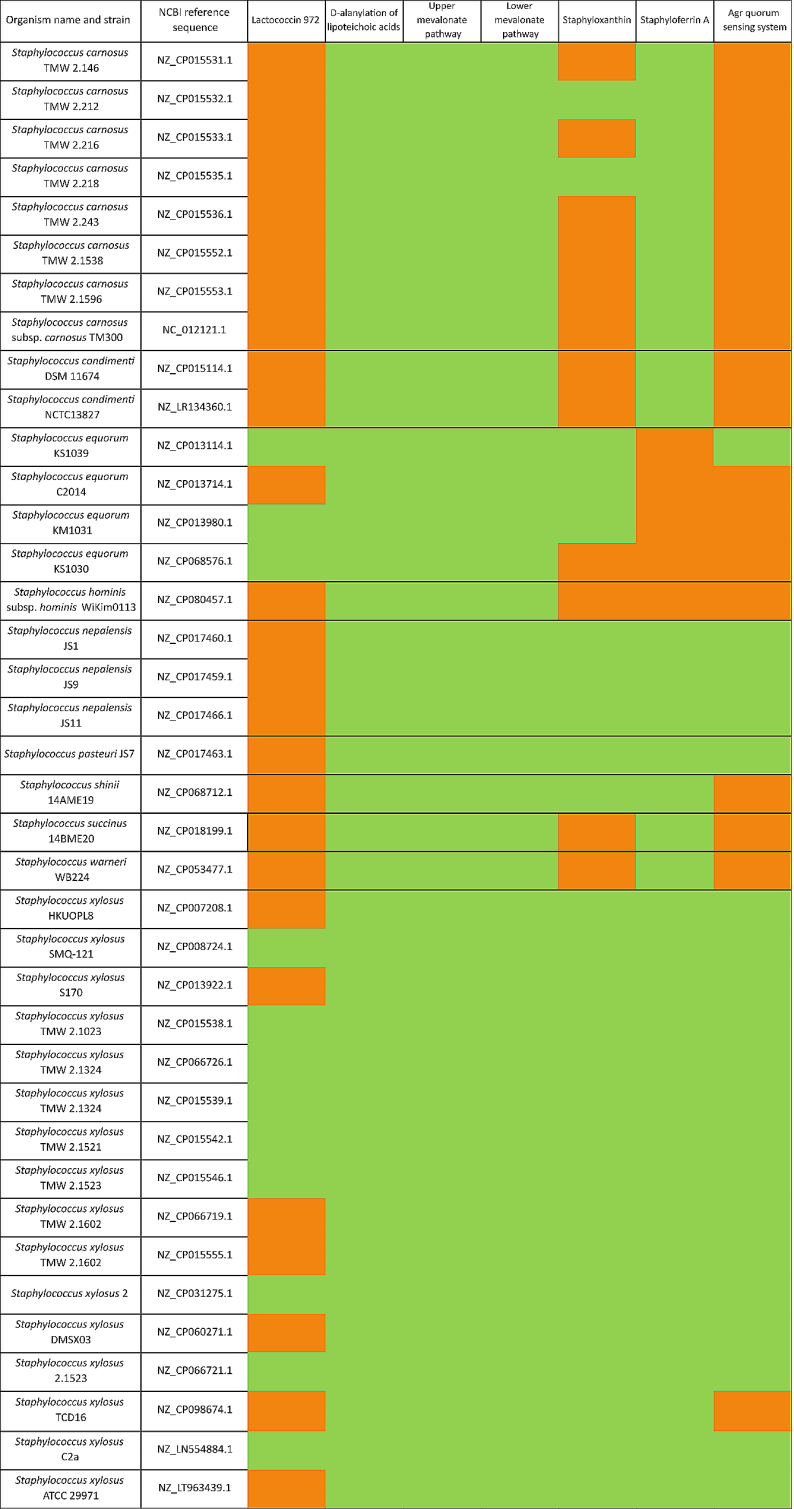
Overview of the presence or absence of the biosynthetic gene clusters (BGCs) present in *Staphylococcus shinii* IMDO-S216 in the bacterial genomes selected for comparative genomics analysis (Table 1). Green boxes: presence of all genes of the BGC. Orange boxes: one or more genes of the BGC are absent. The criteria applied to consider two sequences as homologous are a minimum of 30% sequence identity and a query coverage of at least 70%; the query coverage and percentage identity values for each gene are presented in Table S1

The genome assembly was annotated with Prokka (v1.14.6) [42]. Next, the bacterial version of the tool antiSMASH (antibiotics and Secondary Metabolites Analysis Shell; v6.0) [43] was used to mine the genome data for secondary metabolite biosynthetic gene clusters (BGCs), applying a profile hidden Markov models-based approach. To do so, the detection strictness was set to ‘relaxed’. Analysis of protein sequences with the blastp algorithm [39] was performed on the antiSMASH output to designate and/or confirm the functions predicted. The BGCs were compared with the widely studied *S. aureus* subsp. *aureus* NCTC 8325 strain (BioProject accession number PRJNA57795). In addition, a comparative analysis focusing on the detected BGCs was performed with selected microorganisms from the *Staphylococcus* genus according to the following criteria: *Staphylococcus xylosus* strains with a complete or chromosome assembly level; *Staphylococcus shinii* strains with a complete or chromosome assembly level; other *Staphylococcus* species found in fermented meat matrices with a complete or chromosome assembly level; and other *Staphylococcus* species found in other fermented food matrices with a complete or chromosome assembly level (Table 1). As such, 16 strains of *S. xylosus* were selected; out of non-*xylosus* species, eight *Staphylococcus carnosus*, two *Staphylococcus condimenti*, four *Staphylococcus equorum*, one *Staphylococcus hominis*, three *Staphylococcus nepalensis*, one *Staphylococcus pasteuri*, one *Staphylococcus shinii* (formerly classified as *Staphylococcus pseudoxylosus*), one *Staphylococcus succinus*, and one *Staphylococcus warneri* strain(s) were selected. Except for *S. xylosus* TCD16 and *S. xylosus* ATCC 29,971, with a chromosome assembly level, all strains selected displayed a complete assembly level. Out of the 38 strains, 14 were isolated from fermented sausages, nine of them being *S. xylosus* isolates. Other fermented sources of isolation were fish sauce, soy sauce mash, shrimp paste, kimchi, and soybeans. *Staphylococcus xylosus* strains with a complete or chromosome assembly level were also isolated from animal faecal matter, mouse rectum, leafy vegetable, milker’s hands and, in less detail, human skin. By the time of the selection of these organisms for a comparative analysis, out of the 16 *S. shinii* present in the NCBI Genome database, only *S. shinii* 14AME19 presented a complete assembly level. The other candidate strains, even if they were ecologically relevant due to their sourcing from fermented soybeans, bovine mastitis samples, or raw cow’s milk, only presented contig or scaffold assembly level, and were therefore not selected. The genomes were retrieved from the Genome database of the NCBI (accessed March 2024). The amino acid sequences of the BGCs of interest of *S. shinii* IMDO-S216 were aligned against the nucleotide sequences of the microorganisms selected using the tblastn algorithm [44]. A summarized comparison with the hypothesized presence or absence of the BGCs is displayed in Fig. 1. Two sequences were considered as homologous if their alignment had a minimum sequence identity of 30% and a query coverage of at least 70% [45]. The percentage identity and query coverage of each gene were individually inferred and their probable presence or absence was indicated through a colour code based on the previously mentioned criteria (Table S1).

**Table 1:** Overview of selected bacterial genomes, retrieved from the Genome database of the National Centre for Biotechnology Information (NCBI), for the performance of a comparative analysis

#	Organism name and strain	NCBI reference sequence (chromosome)	Genome assembly (Mb)	GC%	Scaffolds	Coding sequences	Release date	Sequencing methodology	Source	Reference
1	*Staphylococcus carnosus* TMW 2.146	NZ_CP015531.1	2.57	34.7	1	2,392	July 2022	PacBio	Fermented sausage	Direct submission
2	*Staphylococcus carnosus* TMW 2.212	NZ_CP015532.1	2.62	34.5	1	2,446	July 2022	PacBio	Fermented fish sauce	Direct submission
3	*Staphylococcus carnosus* TMW 2.216	NZ_CP015533.1	2.65	34.54	2	2,484	July 2022	PacBio	Fermented fish	Direct submission
4	*Staphylococcus carnosus* TMW 2.218	NZ_CP015535.1	2.64	34.6	1	2,483	July 2022	PacBio	Fermented fish sauce	Direct submission
5	*Staphylococcus carnosus* TMW 2.243	NZ_CP015536.1	2.57	34.7	1	2,391	July 2022	PacBio	Fermented sausage	Direct submission
6	*Staphylococcus carnosus* TMW 2.1538	NZ_CP015552.1	2.62	34.6	1	2,440	July 2022	PacBio	Fermented sausage	Direct submission
7	*Staphylococcus carnosus* TMW 2.1596	NZ_CP015553.1	2.65	34.54	2	2,472	July 2022	PacBio	Fermented sausage	Direct submission
8	*Staphylococcus carnosus* subsp. *carnosus* TM300	NC_012121.1	2.57	34.6	1	2,382	February 2009	DNA capillary sequencer	Fermented sausage	Rosenstein et al. (2009) [46]
9	*Staphylococcus condimenti* DSM 11,674	NZ_CP015114.1	2.66	34.7	1	2,462	April 2016	PacBio	Soy sauce mash	Dong et al. (2017) [47]
10	*Staphylococcus condimenti* NCTC13827	NZ_LR134360.1	2.65	34.7	1	2,452	December 2018		Soy sauce mash	Direct submission
11	*Staphylococcus equorum* KS1039	NZ_CP013114.1	2.82	33.1	1	2,645	November 2015	PacBio; Illumina	Fermented shrimp paste	Jeong et al. (2016) [48]
12	*Staphylococcus equorum* C2014	NZ_CP013714.1	2.93	32.84	6	2,700	July 2016	PacBio; Illumina	Fermented shrimp paste	Direct submission
13	*Staphylococcus equorum* KM1031	NZ_CP013980.1	2.79	33.02	4	2,595	July 2016	PacBio; Illumina	Fermented anchovies	Heo et al. (2022) [49]
14	*Staphylococcus equorum* KS1030	NZ_CP068576.1	2.95	33.17	5	2,814	January 2021	PacBio; Illumina	Salted fermented seafood	Kim et al. (2021) [50]
15	*Staphylococcus hominis* subsp. *Hominis* WiKim0113	NZ_CP080457.1	2.24	31.5	1	2,084	May 2022	PacBio	Kimchi	Direct submission
17	*Staphylococcus nepalensis* JS1	NZ_CP017460.1	2.99	33.02	3	2,794	October 2017	PacBio	Korean fermented food	Direct submission
16	*Staphylococcus nepalensis* JS9	NZ_CP017459.1	2.89	33.1	1	2,676	May 2018	PacBio	Korean fermented food	Direct submission
18	*Staphylococcus nepalensis* JS11	NZ_CP017466.1	3.01	33.07	3	2,775	October 2017	PacBio	Fermented shrimp paste	Direct submission
19	*Staphylococcus pasteuri* JS7	NZ_CP017463.1	2.62	31.55	3	2,466	October 2017	PacBio	Korean fermented food	Direct submission
20	*Staphylococcus shinii* 14AME19	NZ_CP068712.1	3.01	32.69	5	2,729	January 2021	PacBio	Fermented soybean	Kong et al. (2021) [3]
21	*Staphylococcus succinus* 14BME20	NZ_CP018199.1	2.75	33.10	1	2,546	December 2016	PacBio	Fermented soybeans	Jeong & Lee (2017) [51]
22	*Staphylococcus warneri* WB224	NZ_CP053477.1	2.57	32.63	7	2,386	May 2020	PacBio; Illumina	Spicy fermented bean paste	Direct submission
23	*Staphylococcus xylosus* HKUOPL8	NZ_CP007208.1	2.87	32.76	2	2,608	June 2014	454 pyrosequencing	Animal faecal matter	Ma et al. (2014) [52]
24	*Staphylococcus xylosus* SMQ-121	NZ_CP008724.1	2.76	32.90	1	2,490	June 2014	PacBio; Illumina	Fermented sausage	Labrie et al. (2014) [53]
25	*Staphylococcus xylosus* S170	NZ_CP013922.1	2.91	33.00	1	2,688	April 2017	PacBio; Illumina	Leafy vegetable	Direct submission
26	*Staphylococcus xylosus* TMW 2.1023	NZ_CP015538.1	2.8	32.80	1	2,531	July 2022	PacBio	Fermented sausage	Direct submission
35	*Staphylococcus xylosus* TMW 2.1324	NZ_CP066726.1	2.97	32.85	4	2,712	October 2021	Illumina	Fermented sausage	Direct submission
27	*Staphylococcus xylosus* TMW 2.1324	NZ_CP015539.1	2.97	32.85	3	2,709	July 2022	PacBio	Fermented sausage	Direct submission
28	*Staphylococcus xylosus* TMW 2.1521	NZ_CP015542.1	3.01	32.73	4	2,737	July 2022	PacBio	Fermented sausage	Direct submission
29	*Staphylococcus xylosus* TMW 2.1523	NZ_CP015546.1	2.98	32.67	5	2754	July 2022	PacBio	Fermented sausage	Direct submission
33	*Staphylococcus xylosus* TMW 2.1602	NZ_CP066719.1	2.79	32.90	2	2,522	October 2021	Illumina	Fermented sausage	Direct submission
30	*Staphylococcus xylosus* TMW 2.1602	NZ_CP015555.1	2.85	32.88	2	2,569	July 2022	PacBio	Fermented sausage	Direct submission
31	*Staphylococcus xylosus* 2	NZ_CP031275.1	2.85	32.80	2	2,607	August 2019	PacBio	Milker’s hands	Direct submission
32	*Staphylococcus xylosus* DMSX03	NZ_CP060271.1	2.87	32.76	2	2,612	August 2020	PacBio	Fermented soybean	Heo et al. (2021) [54]
34	*Staphylococcus xylosus* 2.1523	NZ_CP066721.1	2.98	32.67	5	2745	October 2021	Illumina	Fermented sausage	Direct submission
36	*Staphylococcus xylosus* TCD16	NZ_CP098674.1	2.76	33.70	1	2,424	June 2022	Illumina	Mouse rectum	Direct submission
37	*Staphylococcus xylosus* C2a	NZ_LN554884.1	2.79	32.90	1	2,551	September 2014	DNA capillary sequencing	Not specified	Direct submission
38	*Staphylococcus xylosus* ATCC 29,971	NZ_LT963439.1	2.78	32.90	1	2,521	April 2018	PacBio; Illumina	Human skin	Direct submission

In parallel, a manual blastp analysis was performed, in which the protein database UniProt [55] was used to retrieve a compilation of the amino acid sequences from the order Bacillales and compared with those of *S. shinii* IMDO-S216 predicted by Prokka. The Conserved Domains Database webportal (CDD) [56] was used to visualize the proteins’ conserved regions. The PredictProtein server [57] was used to get an in-depth look at the structure and function of the proteins of interest. Screening for virulence factors (VFs) was performed through the online platform Vfanalyzer [58] of the Virulence Factor Database (VFDB) under default settings. BAGEL4 [59] was used to detect gene clusters related to bacteriocins and ribosomally synthesized and posttranslationally modified peptides (RiPPs). A summary of the bioinformatic tools used for each genomic cluster discovered is detailed in Table 2. The Artemis tool (v18.2.0) [60] was used to assess the genomic context of each sequence of interest.

**Table 2:** Description of the bioinformatic tools used in this study and the gene cluster(s) of relevance

Name of the tool	Purpose	Genomic cluster(s)
antiSMASH (antibiotics and Secondary Metabolites Analysis SHell) [36]	When using a nucleic acid sequence file as input, this tool mines its secondary metabolite biosynthetic gene clusters (BGCs), providing an initial scaffold for a further study of the microorganism’s genomic islands. With this tool, the six described genomic clusters were found for the studied strain.	Synthesis of lactococcin 972 D-alanylation of lipoteichoic acids Synthesis of mevalonate Synthesis of staphyloxanthin Synthesis of staphyloferrin A Synthesis of the quorum-sensing system
BAGEL4 [44]	When genomic DNA files are used as an input, this tool detects genes encoding bacteriocins and ribosomally synthesized and posttranslationally modified peptides (RiPPs). In this study, however, the only output obtained was the lactococcin 972 gene cluster as a putative bacteriocin-encoding genomic element; this tool was, therefore, used to corroborate the hypothetical function of this element.	Synthesis of lactococcin 972
blastp (Basic Local Alignment Search Tool, protein-protein alignment query) [37]	This tool was used to align every amino acid sequence derived from the genes found in the gene clusters, against protein databases. This allowed to determine the preliminary functions of the studied genes, including information such as the statistical significances of the matches.	Synthesis of lactococcin 972 D-alanylation of lipoteichoic acids Synthesis of mevalonate Synthesis of staphyloxanthin Synthesis of staphyloferrin A Synthesis of the quorum-sensing system
UniProt [40]	In particular gene clusters, such as the one related to the synthesis of mevalonate, blastp was not able to elucidate the nomenclature and function of certain enzymes. To this end, the database UniProt was used to retrieve a compilation of the amino acid sequences from the order Bacillales, and then compared to the amino acid sequences of the studied strain. This allowed to elucidate enzymatic functions that were previously unclear.	Synthesis of mevalonate
CDD (Conserved Domain Database webportal) [41]	Every result obtained from the blastp tool where putative conserved domains were detected was verified in the CDD webportal as well, to obtain further information about the function of the gene and an E-value of the conserved domain.	Synthesis of lactococcin 972 D-alanylation of lipoteichoic acids Synthesis of mevalonate Synthesis of staphyloxanthin Synthesis of staphyloferrin A Synthesis of the quorum sensing system
Artemis tool [45]	This genome browser allowed to assess the genomic context of each gene cluster, as an in-genome visualization method.	Synthesis of lactococcin 972 D-alanylation of lipoteichoic acids Synthesis of mevalonate Synthesis of staphyloxanthin Synthesis of staphyloferrin A Synthesis of the quorum-sensing system

### Illustrations

Gene clusters were represented using the packages ggplot2 (v3.4.1) [61] and gggenes (v0.4.1) [62] in Rstudio (v4.2.2) [63], and biosynthetic pathways were plotted with the use of Inkscape (v1.1.2) [64].

## Results

### General features of the *de novo*-assembled genome of *Staphylococcus shinii* IMDO-S216

The ONT and Illumina sequence read sets contained 117,011 reads with on average 587× coverage and 5,590,125 reads with on average 496× coverage, respectively. The output of the Unicycler assembler in Normal resolution mode was manually curated using the output from Unicycler’s Bold resolution mode, resulting in the *Staphylococcus shinii* IMDO-S216 genome containing nine contigs, i.e., a single circular chromosome of 3.01 Mb and eight plasmid replicons (Table 3). Plasmids pIMDO_S216_8 and pIMDO_S216_9 displayed identical size but pairwise comparison showed 93.80% sequence identity. Also, plasmid pIMDO_S216_9 contained the lincomycin resistance protein (*linA*) and replication protein (*rep*) genes, as well as the quaternary ammonium compound-resistance protein (*qacG*) and replication protein (*rep94*) genes, whereas plasmid pIMDO_S216_8 did not.

**Table 3:** General overview of the genomic composition of *Staphylococcus shinii* IMDO-S216, including genomic features, conformation, size in bp, estimated copy number, and percentage of G + C content

Genomic feature	Conformation	Size (bp)	Estimated copy number (x)	G + C content (%)
Main chromosome	Circular	3,013,896	1.00	32.7
Plasmid pIMDO-S216-1	Linear	128,205	3.30	28.8
Plasmid pIMDO-S216-2	Circular	52,059	1.01	29.0
Plasmid pIMDO-S216-3	Circular	42,884	0.83	30.3
Plasmid pIMDO-S216-4	Circular	4,440	7.90	30.1
Plasmid pIMDO-S216-5	Circular	3,841	10.50	29.6
Plasmid pIMDO-S216-6	Circular	1,960	8.74	30.1
Plasmid pIMDO-S216-7	Circular	2,332	6.31	30.2
Plasmid pIMDO-S216-8	Circular	2,332	10.70	30.0

To explain the presence of several plasmids in a strain, the incompatibility or ‘Inc typing’ system was used, which classifies plasmids by their ability to coexist in a stable manner within the same bacterial strain; this trait is dependent on the strain’s replication machinery. When two co-resident plasmids share the same Inc typing, they use the strain’s same replication mechanisms and are deemed incompatible [65]. The only plasmids found to share typings in the studied strain were pIMDO-S216-1 and pIMDO-S216-8, both typed as IncA/C. The element pIMDO-S216-2 was typed as vapBC, mainly described in literature as part of a toxin-antitoxin system in *Shigella flexneri* [66]. Element pIMDO-S216-3 was typed as IncI1, the most common plasmid type along with IncA/C and commonly isolated from bacteria from human patients and food-producing animals [67]. Element pIMDO-S216-5 was typed as IncHI2, a widely distributed plasmid in *Salmonella* Typhimurium and relevant in its multidrug resistance spread [68]. Element pIMDO-S216-6, typed as IncF, was characterized as highly prevalent in plasmids recovered from *Escherichia coli* of food-producing and companion animals [69]. Elements pIMDO-S216-4 and pIMDO-S216-7 displayed no coincidences in the PubMLST database.

The G + C content of the main chromosome was 32.7%. Based on Prokka, the genome contained 3161 genes, of which 3080 were inferred to be protein coding sequences (CDS); 22 to encode ribosomal RNAs (rRNAs), 58 to encode transfer RNAs (tRNAs), and one to encode transfer-messenger RNA (tmRNA).

Ten secondary metabolite BGCs encoding RiPPs were identified with antiSMASH, of which six were found to produce antagonistic compounds and other competitiveness factors of interest. These BGCs were investigated in more detail, as outlined in the following section. The remaining four clusters followed the same manual curation process and were determined as incomplete, since only the core genes – i.e., genes that are considered essential for the basic biological functions and survival of an organism – were identified, whereas the remaining genes completing the biosynthetic pathways were not found in the vicinity. This led to the conclusion that antiSMASH identified partial gene sequences or duplicated genes which did not correspond to functional/complete clusters.

### Secondary metabolite BGCs identified within the genome of *Staphylococcus shinii* IMDO-S216

#### BGC related to the synthesis of lactococcin 972

**Fig. 2:**
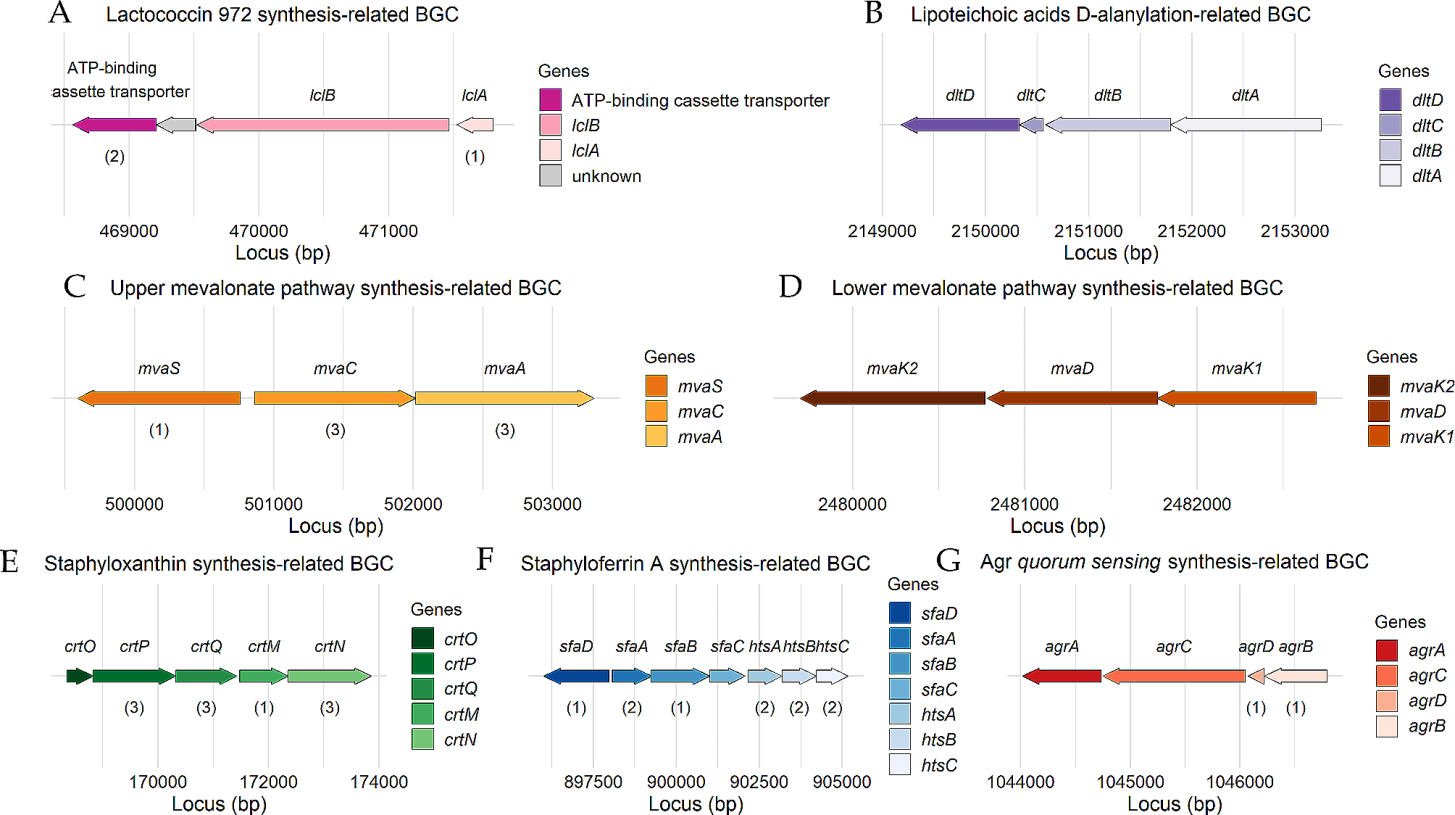
Graphical representation of the *in silico* or manually annotated BGCs belonging to *Staphylococcus shinii* IMDO-S216’s genome. The gene clusters code for: the pre-bacteriocin (*lclA*), the immunity protein (*lclB*), and the ATP-binding cassette transporter, involved in the synthesis of lactococcin 972 (A); the D-alanine-D-alanyl carrier protein ligase (*dltA*), D-alanine-to-teichoic acids incorporation protein and possible transporter (*dltB*), D-alanine-to-carrier transfer protein (*dltC*), and D-alanine transfer protein from the membrane carrier to teichoic acids (*dltD*), involved in the D-alanylation of lipoteichoic acids (B); hydroxy-3-methylglutaryl CoA synthase (*mvaS*), acetyl-CoA acetyltransferase (*mvaC*), and hydroxy-3-methylglutaryl CoA reductase (*mvaA*), which are elements of the upper mevalonate pathway (C); phosphomevalonate kinase (*mvaK2*), mevalonate diphosphate decarboxylase (*mvaD*), and mevalonate kinase (*mvaK1*), which form the lower mevalonate pathway (D); dehydrosqualene synthase (*crtM*), dehydrosqualene desaturase (*crtN*), diaponeurosporene oxidase (*crtP*), glycosyl transferase (*crtQ*), and acyl transferase (*crtO*), necessary for the synthesis of staphyloxanthin (E); D-ornithine synthetases (*sfaBD*), pyridoxal-phosphate-dependent amino-acid racemase (*sfaC*), efflux pump (*sfaA*), solute-binding protein (*htsA*), and a permease component of the ABC transporter (*htsBC*), involved in the synthesis of staphyloferrin A (F); and the quorum sensing system agr formed by the autoinducing peptide (AIP, *agrD*), the AIP proteolytic processor/transporter (*agrB*), peptide sensor kinase (*agrC*), and a transcriptional activator (*agrA*) (G). Some genes were identified by antiSMASH as core biosynthetic (1), transport-related (2), or additional biosynthetic (3)

**Fig. 3:**
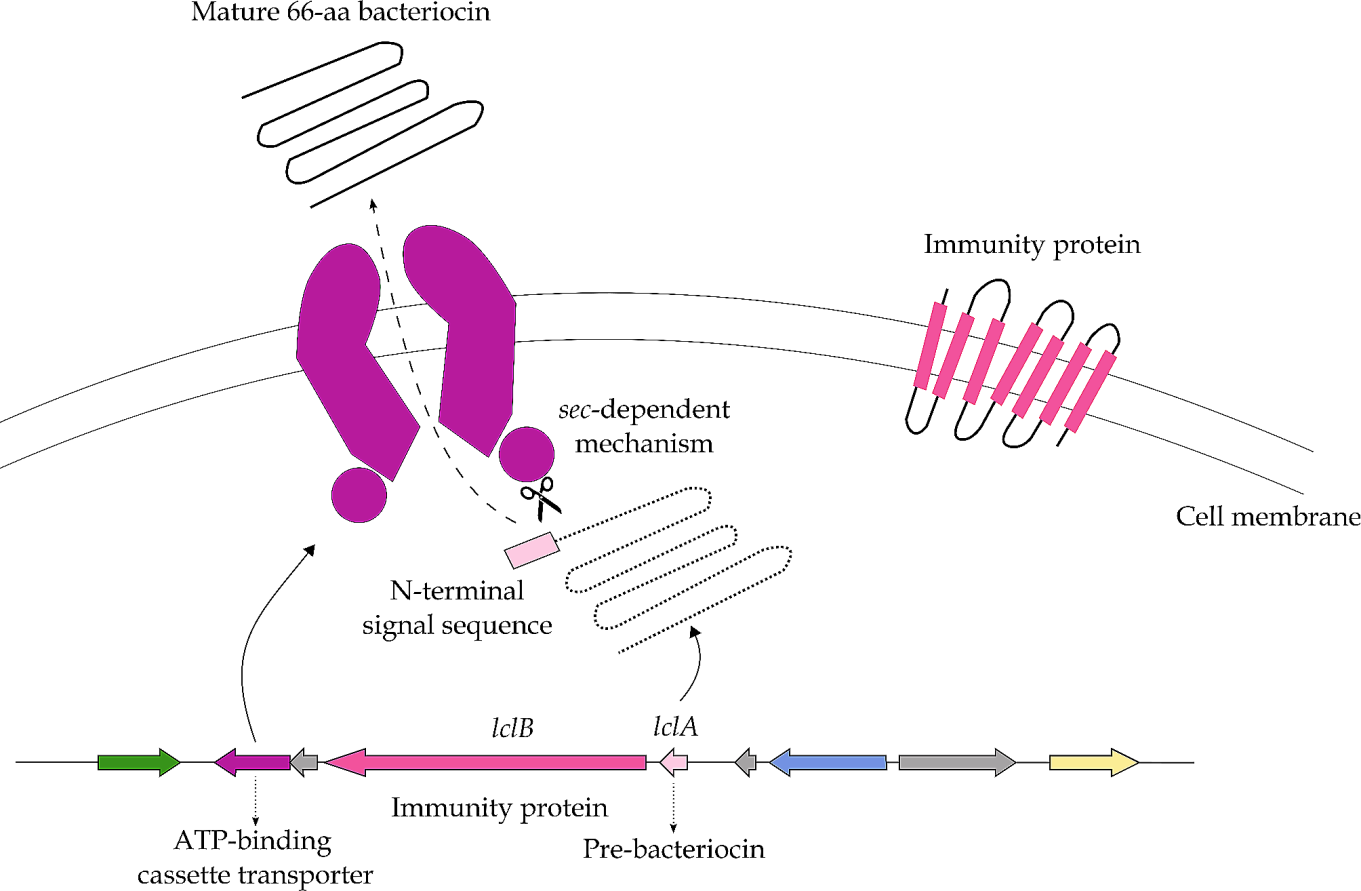
Presumed biosynthesis pathway of lactococcin 972 from its annotated genome cluster. The ABC transporter processes the pre-bacteriocin (LclA) and translocates the product across the plasmid membrane. The immunity protein (LclB) protects the producing bacterium from its bacteriocin’s action. Reconstructed after Stoddard et al. (1992), Klaenhammer (1993), Håvarstein et al. (1995), Venema et al. (1995), Nes et al. (1996), and Nes & Eijsink (1999) [70–75]

The first of the BGCs of interest found by antiSMASH and corroborated by BAGEL4 (Table 2) was located on the reverse strand of the chromosome, spanning a region from position 468,564 to 471,803 (total size of 3,240 nt) and presumably coding for lactococcin 972 (Lcn972) (Fig. 2A). The known biosynthetic pathway of Lcn972 is given in Fig. 3, showing that the pre-bacteriocin of Lcn972, coded for by *lclA*, is synthesized as a precursor peptide that contains an N-terminal extension (leader peptide) which is cleaved off during maturation by the action of an ATP-binding cassette (ABC) transporter. Two conserved glycine residues in the precursor are recognized by the ABC transporter, which has the dual function of removing the leader peptide from the precursor and translocating the processed bacteriocin across the plasmid membrane. The immunity protein, coded for by *lclB*, spans the membrane seven times.

Within the BGC present in *S. shinii* IMDO-S216, the biosynthetic RiPP-like gene was identified as the pre-bacteriocin-encoding gene (*lclA*), immediately preceded by the immunity protein-encoding gene (*lclB*) (Fig. 2A). The encoded pre-bacteriocin protein contained a 23-aa signal peptide. The two conserved glycine residues described above were not found in the leader peptide, although a -GSG- residue sequence was present on positions 17 to 19 from the beginning of the leader peptide, as well as a -GG- residue sequence on positions 31 to 32. At a distance of 300 nucleotides downstream from the immunity protein-encoding gene, a gene coding for an ABC transporter was located; the finding of this gene in the vicinity of the bacteriocin operon confirmed completeness of the cluster. From the comparative analysis, it could be inferred that the presence of a complete lactococcin 972 cluster was not a common trait, occurring only in some *S. equorum* strains and nine of the *S. xylosus* strains (Fig. 1). While the ATP-binding cassette transporter gene was ubiquitous in all genomes, either *lclA*, *lclB*, or both genes did not reach the minimum homology thresholds in 26 of the genomes selected (Table S1).

#### BGC related to the D-alanylation of lipoteichoic acids

Another BGC of interest was located on the reverse strand of the chromosome from position 2,149,180 to 2,153,254 (Fig. 2B). This cluster may be involved in the process of D-alanylation of lipoteichoic acids, a component of the cell wall. The resulting cell wall modification displays effects such as resistance against cationic antimicrobial peptides of human, animal, and microbial origin [76]. Another competitiveness-related effect found consisted of an improved adaptation to acidification, paramount in fermentative environments [77]. The *dltABCD* operon encoded four proteins responsible for the esterification of lipoteichoic acid by D-alanine. In this regard, the product of *dltA* acts as a D-alanine-D-alanyl carrier protein ligase (Dcl) that transfers a D-alanine to a D-alanine carrier protein (Dcp), encoded by *dltC*; the product of *dltB* cooperates in the incorporation of D-alanine into teichoic acids and may play a role in the transfer of D-alanine across the cytoplasmic membrane; and the product of *dltD* is involved in the transfer of D-alanine from the membrane carrier to teichoic acids.

In *S. shinii* IMDO-S216, only one standalone gene of this operon (*dltA_1*) was identified by antiSMASH (data not shown). The other genes (*dltBCD*) were not found in the vicinity of the cluster. Through manual curation, a complete *dltABCD* gene cluster was found at the position mentioned above (Fig. 2B). A second standalone *dltA* gene (*dltA_2*) was found on the chromosome at position 32,608 to 41,679 (data not shown). Whereas *dltA* was coding for a protein of 487 amino acids, the *dltA_1* and *dltA_2* genes were encoding proteins of 1442 and 3023 amino acids, respectively. A manual alignment using blastp revealed that the amino acid sequence encoded by *dltA* had 95% sequence coverage with a 483- and 472-amino acid section of the sequences encoded by *dltA_1* and *dltA_2*, respectively; *dltA* shared, in addition, around 30% of sequence identity with both standalone genes. The aligning regions of both *dltA_1* and *dltA_2* with *dltA* were located from amino acid position 458 to 941 and amino acid position 482 to 954, respectively. These locations confirm that the presence of homology is prone to be caused by a duplication of the gene and not due to the loss of a stop codon, in which case the homologous regions would be probably located at the beginning of the *dltA* sequence. The shared conserved region between these homologous genes was an adenylate-forming domain of around 478 nt long. This cluster was complete and present in each of the 38 strains selected for the comparative analysis, with all genes having a substantially higher percentage of identity in all *S. xylosus* strains and in *S. shinii* (Fig. 1; Table S1).

#### BGC related to the synthesis of mevalonate

A gene cluster was inferred to code for the upper mevalonate pathway, located on the chromosome from position 499,590 to 503,300 (total size of 3,711 nt) (Fig. 2C). Mevalonate is one of the central molecules of the biosynthetic pathway of isoprenoids, a pathway that is not only highly conserved through evolution and widely spread through all life forms, but also the origin of some of the most ancient biomolecules identified [78]. Some of the key molecules derived from isoprenoids are, for example, undecaprenyl pyrophosphate, a fundamental element of the cell wall peptidoglycan, as well as menaquinones and ubiquinones, required as electron carriers in the generation of respiratory energy [79].

**Fig. 4:**
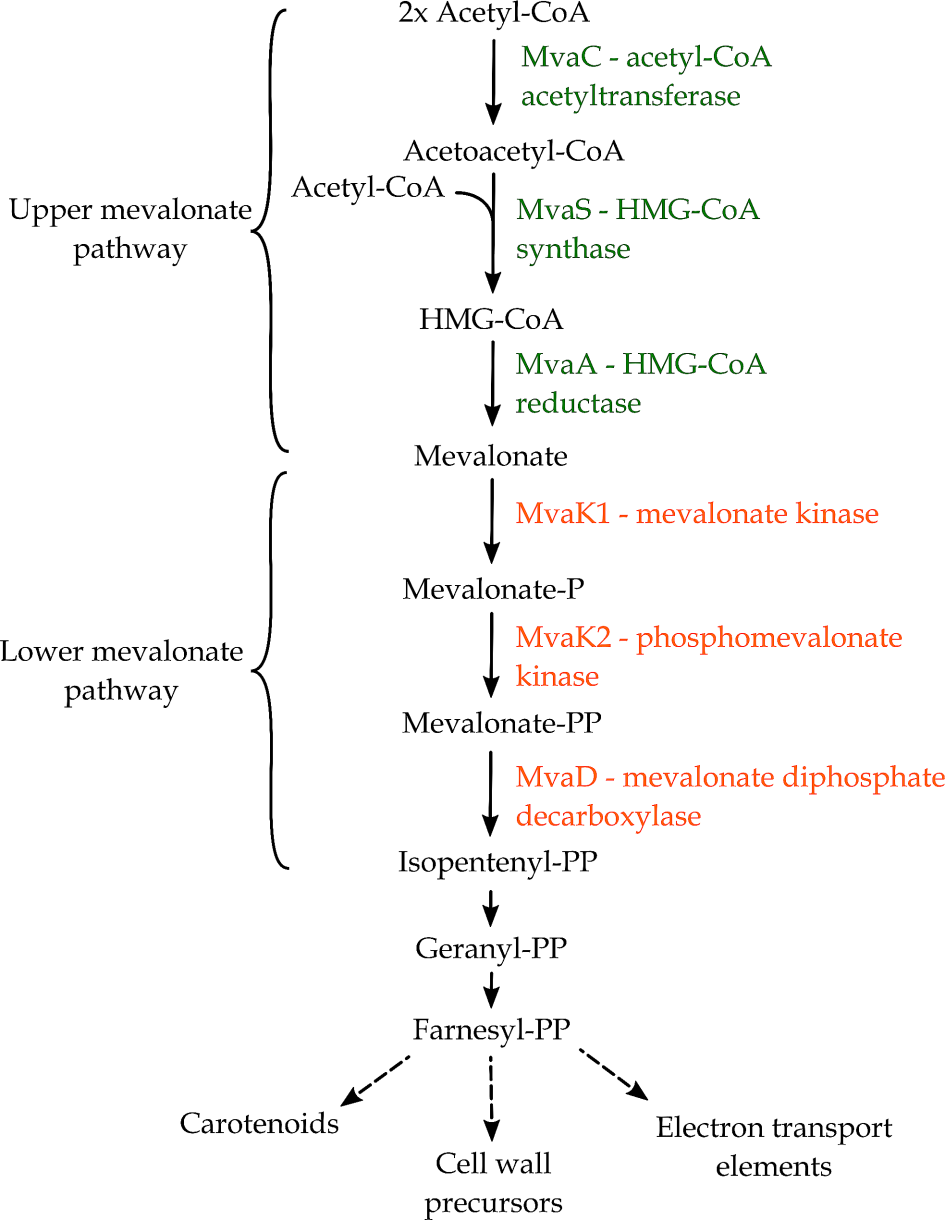
Predicted biosynthetic pathway of isoprenoids and their by-products in bacteria, after Wilding et al. (2000), Tsubakishita et al. (2010), and Reichert et al. (2018) [80–82]. Depicted in green are the enzymes found in the gene cluster identified by antiSMASH, while the enzymes found through manual curation with the use of UniProt and blastp are depicted in orange. HMG-CoA: hydroxy-3-methylglutaryl coenzyme A

In staphylococci, six enzymes comprise the mevalonate pathway consisting of both an upper and lower pathway (Fig. 4). The first three enzymes catalyse the conversion from acetyl-CoA to mevalonate (upper pathway). The process starts with three acetyl-CoA units joined consecutively: first through acetyl-CoA acetyltransferase encoded by *mvaC*, followed by hydroxy-3-methylglutaryl coenzyme A synthase encoded by *mvaS* to form hydroxy-3-methylglutaryl coenzyme A (HMG-CoA), which is then reduced to mevalonate through the action of HMG-CoA reductase (*mvaA*). In the lower pathway, of which the genes were encoded at another location on the chromosome, namely from position 2,479,692 to 2,482,693 (Fig. 2D), mevalonate is phosphorylated and decarboxylated through the enzymes mevalonate kinase (*mvaK1*), phosphomevalonate kinase (*mvaK2*), and mevalonate diphosphate decarboxylase (*mvaD*), resulting in isopentenyl diphosphate (IPP). Both upper and lower mevalonate pathways were present in each strain considered in the comparative analysis, sharing 84.8 to 98.97% and 88.45 to 100% of sequence identity across all genes for all *S. xylosus* and *S. shinii* strains, respectively (Table S1).

#### BGC related to the synthesis of staphyloxanthin

A gene cluster, located from position 168,352 to 173,857 (total size of 5,506 nt) on the forward strand of the chromosome (Fig. 2E), was inferred to be related with the synthesis of the yellow-orange carotenoid staphyloxanthin, as it contained the *crtOPQMN* operon. Production of the carotenoid staphyloxanthin reduces susceptibility to killing by hydrogen peroxide and promotes survival of *S. aureus* in the presence of oxidants [83]. Moreover, a decrease in the production of staphyloxanthins has an effect on membrane fluidity and susceptibility to membrane-targeting antibiotics [84].

**Fig. 5:**
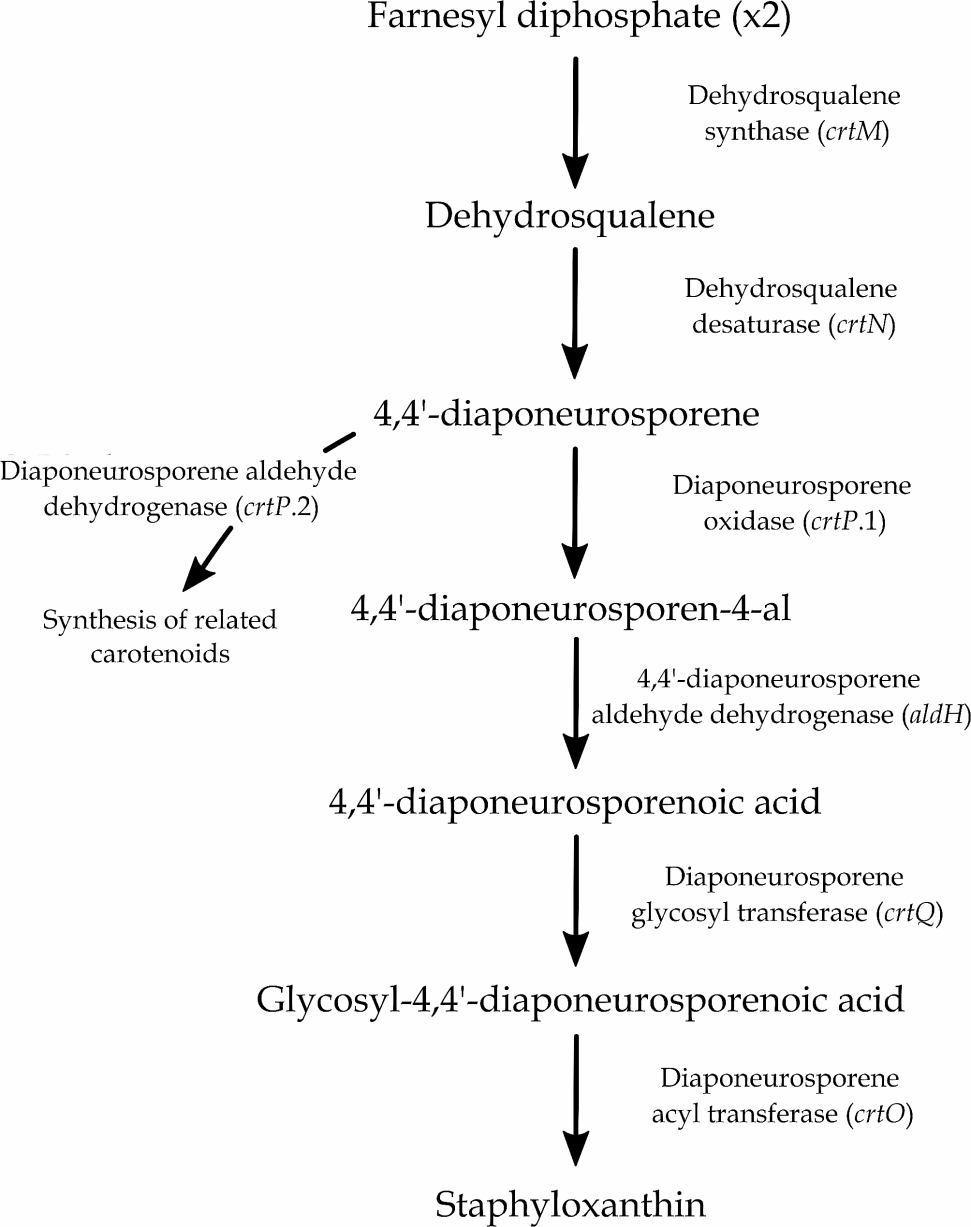
Proposed pathway of staphyloxanthin biosynthesis in *Staphylococcus shinii* IMDO-S216 based on the *in silico* annotation of the BGC, after Pelz et al. (2005), Kim & Lee (2012), and Yehia et al. (2022) [85–87]

The synthesis starts with the condensation of two molecules of farnesyl diphosphate into dehydrosqualene, catalysed by the dehydrosqualene synthase CrtM. This molecule then undergoes dehydrogenation by the dehydrosqualene desaturase CrtN to form 4,4’-diaponeurosporene, which finally undergoes some transformations consisting of oxidation by the diaponeurosporene oxidase CrtP via an aldehyde intermediate, glycosylation by the glycosyl transferase CrtQ, and esterification by the acyl transferase CrtO to produce staphyloxanthin. However, to have a fully operational pathway, a gene encoding a 4,4’-diaponeurosporene aldehyde dehydrogenase (*aldH*) was missing in the initial genome annotation (Fig. 5). Manual alignment of the amino acid sequence of *aldH* from the genome of *S. aureus* subsp. *aureus* NCTC 8325 to the genome sequence of *S. shinii* IMDO-S216 showed the presence of a gene encoding 4,4’-diaponeurosporene aldehyde dehydrogenase (*aldH1*), located from position 1,068,755 to 1,070,134, of which the related amino acid sequence had a 73% sequence identity and a 99% sequence similarity with *S. aureus* subsp. *aureus* NCTC 8325 as the query sequence. The comparative analysis showed that all 16 strains of *S. xylosus*, along with *S. shinii*, *S. pasteuri*, *S. nepalensis*, several *S. equorum* and two out of eight *S. carnosus* contained the complete BGC in their genomes. All *S. warneri*, *S. succinus*, *S. hominis*, *S. condimenti*, one *S. equorum*, and most *S. carnosus* strains lacked at least one gene. The genomes of *S. equorum* KS1030, *S. hominis* subsp. *hominis* WiKim0113, and *S. succinus* 14BME20 specifically lacked all *crtOPQMN* genes, with the *aldH* gene being the only one ubiquitous present in all genomes studied. In genomes lacking one or more elements of this BGC, the genes *crtO* and *crtP* were found in most cases to not reach minimum homology thresholds against the genome under study, as was the case for *S. succinus*, *S. equorum* KS1030, all *S. condimenti*, and most *S. carnosus* strains (Fig. 1; Table S1).

#### BGC related to the synthesis of staphyloferrin A

**Fig. 6:**
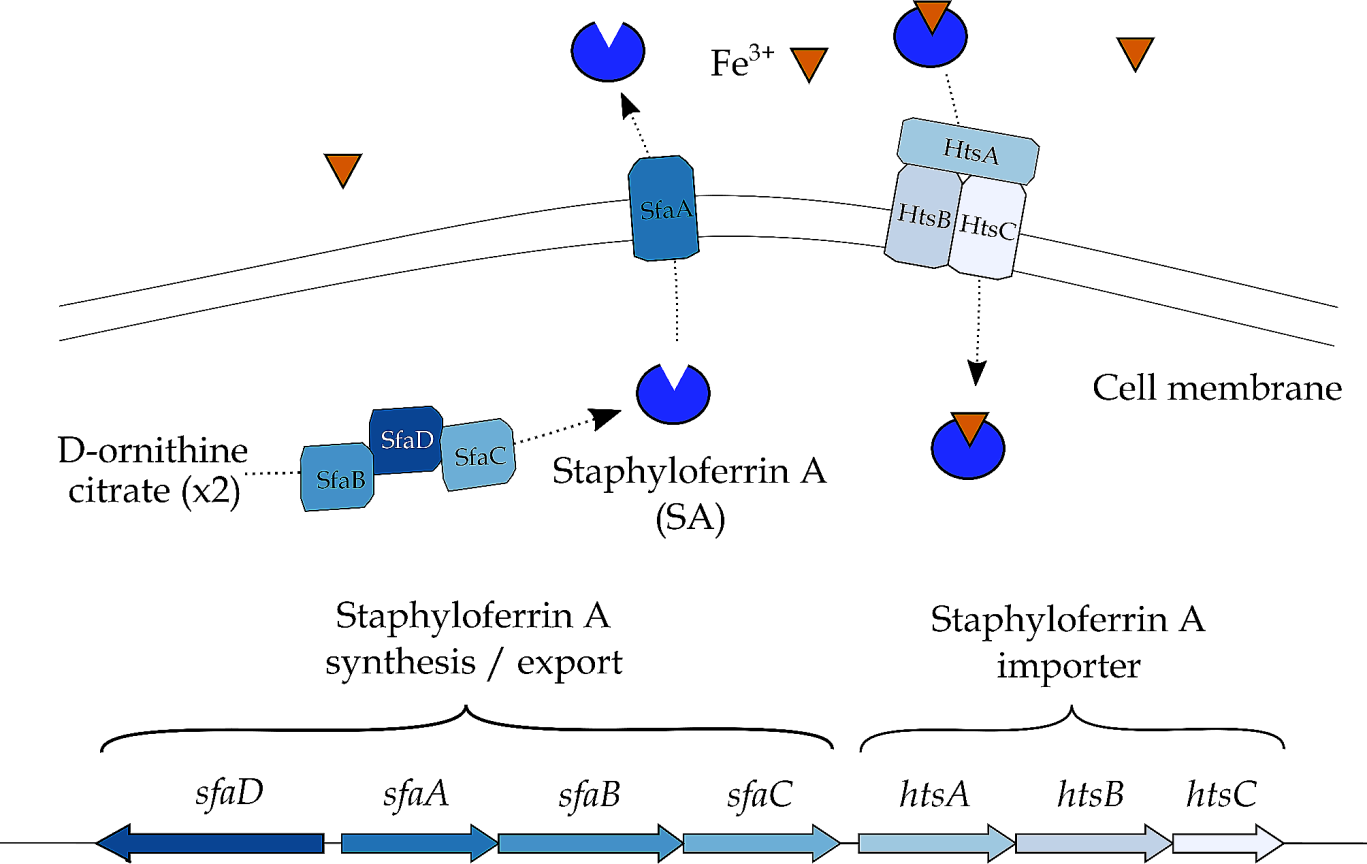
Presumed biosynthetic pathway of staphyloferrin A as described in *Staphylococcus aureus*, after Beasley et al. (2009), Cheung et al. (2009), Beasley & Heinrichs (2010), Laakso et al. (2016), Flannagan et al. (2022), and Van Dijk et al. (2022) [88–93]

A gene cluster, located on the chromosome from position 896,013 to 905,180 (total size of 9,168 nt) (Fig. 2F), was hypothesized to be involved in the production of staphyloferrin A (SA). In the genome of *S. aureus* subsp. *aureus* NCTC 8325, seven genes are related to the biosynthetic process of this siderophore, which are present in two different loci, located next to each other in the genome (Fig. 6). The first locus in the studied strain consists of the genes *sfaDABC*, of which the genes *sfaB* and *sfaD* encode two synthetases that catalyse the condensation of D-ornithine with two molecules of citrate, *sfaC* encodes a pyridoxal-phosphate-dependent amino-acid racemase, and *sfaA* encodes an efflux pump that secretes SA extracellularly. The second locus contains the genes *htsABC*, of which *htsA* encodes a predicted solute-binding protein, and *htsBC* encode a predicted permease component of the ABC transporter required for the uptake of SA.

In the genome of *S. shinii* IMDO-S216, the two genes identified as core genes corresponded with *sfaD* and *sfaB*. Located between *sfaD* and *sfaB*, *sfaA* was found. The gene *sfaC* was located contiguously to *sfaB*. Following this locus, the next three genes constituted the *htsABC* locus and were located downstream, adjoining the *sfaDABC* cluster. This BGC was widely present in most genomes considered in the comparative genomics analysis (Fig. 1). The only lacking element was the gene *sfaA*, which had no ortholog present in the genomes of *S. equorum* KS1039, KM1031, and KS1030, and *S. hominis* subsp. *hominis* WiKim0113, whereas it displayed a very low query coverage with the genome of *S. equorum* C2014 (Fig. 1; Table S1).

This BGC was fairly challenging to identify since the gene identities of this cluster were very similar to the ones producing the siderophores aerobactin and staphyloferrin B, although their biosynthetic processes and gene orientations differed.

#### BGC related to the synthesis of the accessory gene regulator (Agr) quorum sensing system

**Fig. 7:**
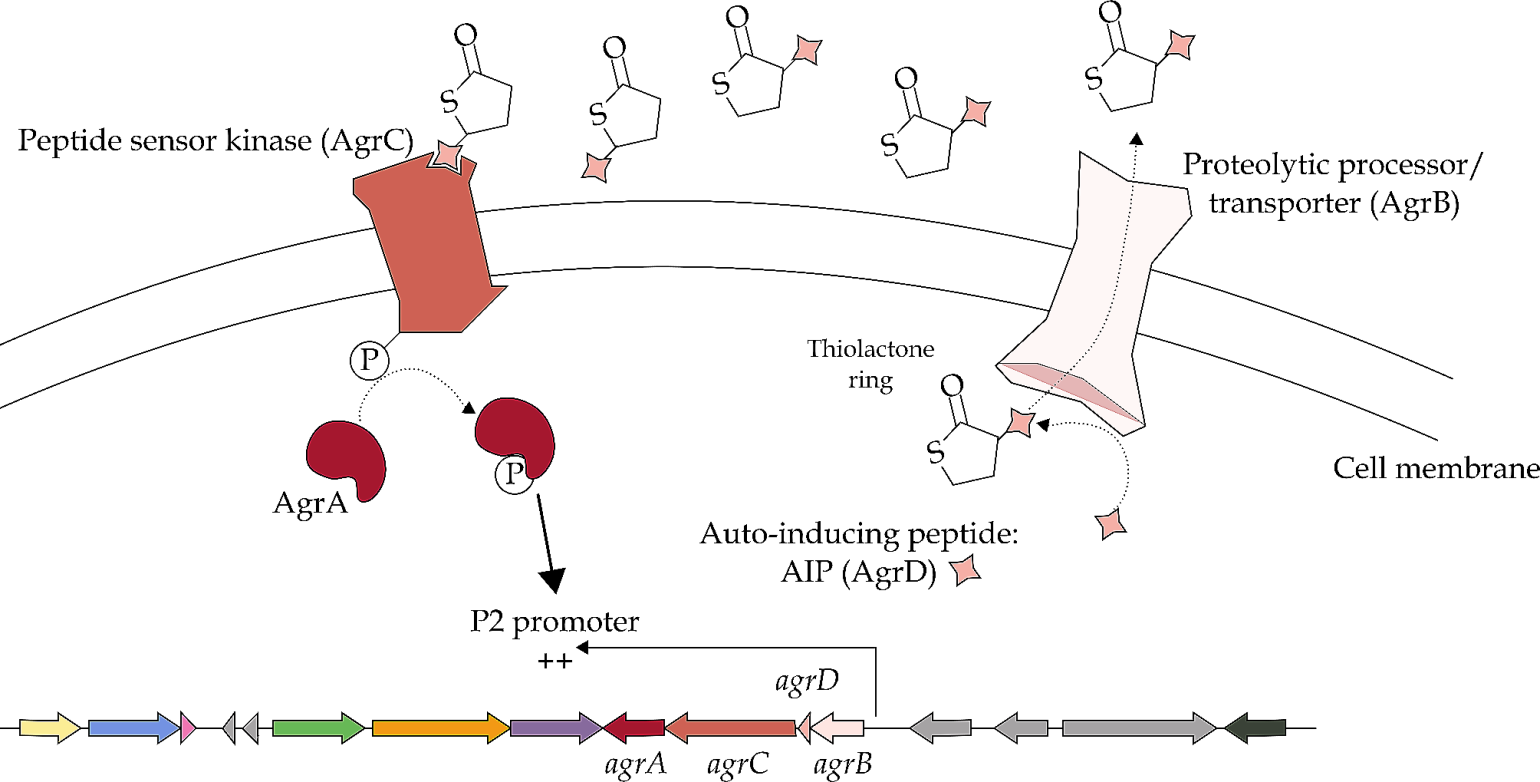
Presumed mode of action of *S. shinii* IMDO-S216’s quorum sensing mechanism, the staphylococci’s accessory gene regulator (Agr) system. The autoinducing peptide (AgrD) is modified and exported by a proteolytic processor/transporter (AgrB); when surpassing a concentration threshold, the peptide sensor kinase (AgrC) triggers a phosphor transfer, which upregulates the transcription of the *agr* operon through the P2 promoter. The pathway was reconstructed after Janzon & Arvidson (1990), Reading & Sperandio (2006), and Verdon et al. (2009) [94–96]

A BGC presumably involved in a bacterial quorum sensing system was located on the reverse strand of the chromosome from position 1,046,792 to 1,044,021 (total size of 2,772 nt) (Fig. 2G). This quorum sensing system has been characterized in *S. aureus* and contains two different loci – the *agrBDCA* gene cluster and the *hld* gene (Fig. 7). In general, the autoinducing peptide (AIP) is encoded by the *agrD* gene. AgrB, characterized as both a proteolytic processor of the AIP and as a transporter, adds a thiolactone ring to AIP and exports it out of the cell. When a threshold concentration is reached, the AIP is detected by the peptide sensor kinase AgrC, which then transfers a phosphate to AgrA, a transcriptional activator. In *S. aureus*, it activates promoters P2 and P3, resulting in the expression of two transcripts, one related to the *agrBDCA* gene cluster, the other related to the *hld* gene encoding the δ-hemolysin. Whereas the full *agrBDCA* gene locus was found, the *hld* gene was not present. This gene was however found in twelve of the 38 strains selected for the comparative analysis, with an identity percentage of 64.10% and a query coverage of 88% on all eight *S. carnosus* strains and both *S. condimenti* strains, as well as an identity percentage of 80.65% and a query coverage of 88% on *S. pasteuri* JS7, and an identity percentage of 77.14% and a query coverage of 79% on *S. warneri* WB224 (results not shown). This quorum sensing system was found to be incomplete in the genomes of all *S. carnosus*, all *S. condimenti*, *S. homini*, *S. shinii*, *S. succinus*, and *S. warneri* strains, and most *S. equorum* strains, as well as in *S. xylosus* TCD16. The low query coverage of the gene *agrD* was the reason of the incompleteness of the BGCs, except for *S. warneri* WB224, where the gene *agrC* was found to be non-homologous, and *S. xylosus* TCD16, which was also lacking *agrC* and did not contain an ortholog for *agrD* (Fig. 1; Table S1).

## Discussion

As studied in context with *Staphylococcus shinii*, *Staphylococcus xylosus* is one of the most important starter culture species in meat fermentation processes based on its technological properties and its relatively high adaptation to the fermentative environment [9, 17]. In this study, a genome sequencing approach was followed with the aim of revealing key fitness features of a selected *S. shinii* strain IMDO-S216, formerly classified as *S. xylosus* IMDO-S216, isolated from fermented meat.

Before entering into a more detailed discussion of the factors involved in survival and competitiveness, some limitations of this study need to be specified. First of all, the exploration of competitiveness-enhancing secondary metabolites was based on the use of the antiSMASH tool, to find secondary metabolite BGCs within the strain’s genome. This engine applies validated ‘rules’ to define which core biosynthetic functions need to be present in a genomic region for it to constitute a BGC. Even if antiSMASH is a widely used tool for genome mining, this approach comes with the limitation of not being exhaustive. More detailed genomic studies may bring different competitive elements to light, as could well be the case with antibiotics, lantibiotics, and bacteriocins. These antimicrobial elements have been described in other species of the genus *Staphylococcus*. Some examples include the lantibiotic epidermin, identified in *Staphylococcus epidermidis* [97]; the lantibiotic gallidermin, by *Staphylococcus gallinarum* [98]; and the antibiotic lugdunin, by *Staphylococcus lugdunensis* [99]; or bacteriocins such as β-haemolysin, produced by *S. lugdunensis*; the antimicrobial peptides aureocins A53 and A70, by *Staphylococcus aureus*; lysostaphin, produced by *Staphylococcus simulans* biovar *staphylolyticus*, or its homologous ALE-1 from *Staphylococcus capitis* [100]; the bacteriocin hyicin 3682, detected in *Staphylococcus agnetis* 3682; the lantibiotic nisin J, homologous to nisin A, present in *S. capitis* APC2923; lantibiotic nukacins 3299, L217, and KQU-131, by *Staphylococcus simulans*, *Staphylococcus chromogenes*, and *Staphylococcus hominis*, respectively; or the antibiotic micrococcin P1, produced by strains from distinct bacterial genera, including staphylococci [98, 101]. Other antibiotics that have been originally isolated from other microorganisms can also be produced by staphylococci, as could be the case for tyrocidine, gramicidin A, or gramicidin S, among others [102, 103]. Furthermore, it is important to bear in mind that *in silico* prediction and annotation do not yield information related to gene expression under the conditions of interest.

A key secondary metabolite BGC found in the completed genome of *S. shinii* IMDO-S216 revealed the machinery needed to produce a bacteriocin, namely lactococcin 972 (Lcn972). The latter was first isolated from a *Lactococcus lactis* subsp. *lactis* IPLA 972 strain obtained from a home-made cheese [104, 105], after which it was shown that the bacteriocin-encoding gene cluster was situated on a plasmid [106]. A presumed biosynthesis pathway for the synthesis of Lcn972 in *S. shinii* IMDO-S216 was found based on its presence in *L. lactis* subsp. *lactis* IPLA 972 [106]. The identities of the Lcn972 gene cluster elements were compared to those of the *Lactococcus* genus: the *lclA* gene displayed an identity percentage of 32.10% and a query coverage of 84% against *L. lactis* subsp. *lactis* IPLA 972; the *lclB* gene displayed an identity percentage of 25.19% and a query coverage of 37% against *L. lactis* subsp. *lactis* IPLA 972; and the ABC transporter displayed an identity percentage of 32.41% and a query coverage of 93% against *L. lactis* subsp. *lactis* 972. As described previously, the *lclA* and ABC transporter gene sequences could be considered as homologous, since their alignment had a minimum sequence identity of 30% and a query coverage of at least 70% [45]. Hypothetically, the fact that a strain previously classified as *S. xylosus* contains a gene cluster originally identified in a *L. lactis* subsp. *lactis* strain could possibly be related to horizontal gene transfer (HGT) within the ecological niche of the teat skin and milk environment, as it is inhabited by both bacterial species [107–110], but further studies contextualizing the genomic surrounding of this cluster may be necessary to confirm this hypothesis. In *S. equorum* KS1039, a strain proposed as a potential starter culture for the fermentation of high-salt foods [48], the complete lactococcin 972 gene cluster was as well identified and its expression was experimentally confirmed through the observation of inhibition halos against *S. aureus* RN4220; the pre-bacteriocin-coding gene of the *S. equorum* KS1039 strain displayed 70.97% sequence identity at the amino-acid level with that of *S. shinii* IMDO-S216, on a 100% query cover.

On another note, only specific strains of *S. equorum* and *S. xylosus* contained the complete cluster related to the production of lactococcin 972, possibly due to the close relatedness of these two species within *Staphylococcus* phylogeny [111] (Fig. 1; Table S1).

In its mature state, Lcn972 acts bacteriostatically by blocking septum formation in dividing cells [105, 112, 113]. Its range of activity is fairly narrow, mostly targeting lactococcal strains [106], as is the case for most lactococcal bacteriocins other than lantibiotics [114]. Most other Gram-positive bacteria tested were not affected, including strains belonging to the genera *Bacillus, Clostridium*, *Enterococcus*, and *Listeria*. Likewise, *S. shinii* IMDO-S216, previously described as *S. xylosus* IMDO-S216, was not able to inhibit *C. botulinum* strains in an extensive screening for anticlostridial potential among staphylococci [20]. Nonetheless, even if a starter culture is not able to inhibit common pathogens through its bacteriocin, the latter may still be beneficial because of the potential to improve competitiveness over the background microbiota. Bacteriocinogenic strains have, after all, been studied for their potential to create hurdles in meat and meat products, extending their shelf-life and enhancing food safety [115].

A second BGC coding for a competitiveness factor was related to the D-alanylation of lipoteichoic acids (LTAs), which are cell-wall polymers composed of glycerolphosphate (GroP) polymers with a glycolipid anchor. GroPs can be substituted by D-alanine, which modulates the cell constituent’s polyanionic nature [116, 117]. The consequences of this modification are extensive; mutational studies in diverse bacteria have demonstrated that D-alanylation of LTAs plays a fundamental role in the regulation of autolysis, biofilm formation, and – in the case of *S. aureus* and *Listeria monocytogenes* – virulence mechanisms, among other functions [118–120]. Another described role of the D-alanylation of LTAs is an increased tolerance to low pH and acidification processes, as shown by the up-regulation of the complete gene cluster in strain *S. xylosus* C2a during growth in meat models [77]; the four genes involved showed a 96.07%, 96.15%, 96.78%, and 93.63% sequence identity against those of *S. shinii* IMDO-S216, respectively, on a 100% query cover. The relevance of the action of this BGC may be the reason why it was found in the genomes of all 38 *Staphylococcus* strains studied (Fig. 1; Table S1). Moreover, this modification provides resistance to staphylococci against cationic antimicrobial peptides produced by animals and humans, as well as by competing microorganisms [121]. The expression of this genomic cluster may affect how bacteria behave in the gut after ingestion of fermented foods, as a lack of LTA D-alanylation may parallel lower survival rates after gastric transit and a hypothetically lower in vivo biofilm formation [122]. Additionally, the *dltABCD* operon has been identified in several genera [123, 124]. Even if it is not known to which degree the finding of a complete *dltABCD* gene cluster in the genome of *S. shinii* IMDO-S216 provides an actual fitness advantage during meat fermentation, it is likely to assume some relevance given the above. The discovery of two standalone genes *dltA_1* and *dltA_2* merits some discussion. It has generally been considered as a rule of thumb that two sequences are homologous if they are more than 30% identical over their entire lengths [45]. The common conserved region of *dltA_1* and *dltA_2* belongs to the superfamily of adenylate-forming enzymes, which play a role in a wide range of biological processes, among which ATP-driven adenylation of D-alanine encoded by *dltA* [125, 126]. The presence of this conserved domain in different genomic locations could be related to the adenylate-forming enzyme function in diverse cell processes. In other words, whereas *dltA* codes for D-alanylation of lipoteichoic acids, related activities may be offered in other biological contexts under different conditions by *dltA_1* and *dltA_2*.

Another competitiveness feature encoded in the genome of *S. shinii* IMDO-S216 was related to the biosynthetic pathways of isoprenoids, which have a large range of biological functions [80]. One of the central molecules of these pathways, mevalonate, is the precursor of compounds such as isopentenyl diphosphate (IPP) and farnesyl diphosphate (FPP), which in turn are transformed into monoterpenes, sterols, carotenoids, and ubiquinones, to name a few [81]. The function of these compounds includes antibacterial activity, the formation of membrane and cell wall elements, membrane fluidity, protein translation, and electron transport mechanisms, which all increase the bacterium’s adaptability to its environment [81]. Furthermore, some compounds derived from isoprenoids, such as carotenoids, are considered bioactive compounds, associated to health benefits related to immunity, bone, skin, eye and cardiovascular health, among others [127]. The presence of the upper and lower mevalonate biosynthetic gene clusters is highly conserved across all organisms through evolution [80], in line with the results obtained from our comparative analysis (Fig. 1; Table S1). The strain *S. xylosus* C2a displayed an up-regulation of the complete mevalonate pathway-coding gene cluster involved in the synthesis of FPP, a precursor of carotenoid pigments, when grown in meat models [77]. When alignments were performed, *S. xylosus* C2a’s *mvaSCAK2DK1* genes displayed 98.97%, 97.4%, 96.02%, 98.88%, 96.35%, and 98.05% of amino-acid level sequence identity with the respective genes of *S. shinii* IMDO-S216 on a 100% query cover. The isoprenoid by-product staphyloxanthin may serve to some degree as a competitiveness factor. This orange pigmentation and C30 carotenoid membrane-bound pigment is typical for *S. aureus* and used as a main factor to distinguish it from *Staphylococcus epidermidis* (formerly *Staphylococcus albus*) [85, 128]. Due to its conjugated double bonds, it participates in host infection and evasion of immune system in *S. aureus* through the deactivation of reactive oxygen species produced by macrophages and host neutrophils, as well as protecting lipids, proteins, and DNA, being considered as a biological antioxidant and virulence factor in this species [86, 129–131]. For *S. xylosus*, staphyloxanthin seems to play a role in the fluidity of the membrane when grown at low temperatures and in cell survival after freeze-thaw processes [132]. The presence of the *aldH* gene, described through mutational studies and located around 900 kilobase pairs from the functional gene cluster, seems to complete the enzymatic pathway necessary for the synthesis of the molecule; it has similarly been located in literature hundreds of kilobase pairs away from its respective staphyloxanthin gene cluster [87]. Furthermore, due to CrtP acting as both an oxidase and an aldehyde dehydrogenase [87], this enzyme may be implicated in the synthesis of other structurally or functionally related carotenoids. Based on the various results of the comparative analysis of *Staphylococcus* strains, from a presence of the complete gene cluster in each *S. xylosus* strain to the variable presence in the *S. equorum* strains considered (Fig. 1; Table S1), it could be suggested that individual species present a wide trait variation that may translate in differences in community pigmentation [133]. In *S. xylosus* C2a, up-regulation of the genes involved in the synthesis of FPP during growth in meat models was followed by an up-regulation of the *crtPQMN* gene cluster, involved in the carotenoid biosynthesis pathway from FPP, which could explain why this strain produces a yellow pigment when grown on agar medium [77]; amino-acid sequence identities of 89.52%, 80.69%, 79.86%, and 94.20% were found with the respective *crtPQMN* genes of *S. shinii* IMDO-S216. To the authors’ knowledge, informational sources that explain the presence of the staphyloxanthin gene cluster in other bacterial species than *S. xylosus* and *S. equorum* are not yet available in literature.

Another gene cluster related to competitiveness factors encountered in *S. shinii* IMDO-S216 was that of staphyloferrin A and may relate to iron acquisition. To overcome iron limitation in the environment, microorganisms possess high affinity iron (III) chelator systems called siderophores, which can be synthesized by molecular platforms called non-ribosomal peptide synthetases (NRPS) in a NRPS-dependent system, or in parallel, through an NRPS-independent siderophore (NIS) synthetase system that uses condensation reactions to form the final siderophore structure [134]. Staphyloferrin A is a dicitrate-based siderophore that is synthesized through a NIS system [135], which is not alien to *S. xylosus*. The presence of the complete cluster may thus offer *S. shinii* IMDO-S216, similarly as for *S. xylosus*, the necessary machinery for the synthesis and utilization of this siderophore system. This, in turn, might provide an enhanced acquisition of iron from the medium in meat matrices, thereby enhancing growth and survival. The presence of siderophores can also be useful in other fermented foods, such as cheese, enabling the desired microorganisms to compete effectively for the necessary iron [136]. In meat models, the expression of the staphyloferrin A synthesis-related gene cluster underwent an up-regulation in *S. xylosus* C2a [9]; the *sfaDABChtsABC* gene cluster of *S. xylosus* C2a displayed degrees of identity from 89.09 to 98.12% to that of *S. shinii* IMDO-S216. Additionally, this BGC was present in nearly all species considered in the comparative analysis, excepting for *S. equorum* and *S. hominis* (Fig. 1; Table S1). The presence of other siderophores involved in iron uptake, such as lactoferrin and ferritin, in *S. hominis* has been hypothesized in literature, whereas no information about *S. equorum*’s iron transport systems was found [137]. Other iron transport systems have been described in *S. xylosus* C2a in addition to the Sfa and Hts systems described above, such as Sit, Fhu, and Sst [9]. Finally, an Agr quorum sensing system was identified. The term quorum sensing has been collectively designated for those mechanisms through which bacteria regulate gene expression via cell density. Three main quorum sensing systems have been described, one being specific for Gram-negative bacteria, another being specific for Gram-positive bacteria, and a third system that is considered universal [94]. The Gram-positive quorum sensing mechanism usually involves small peptides that are modified and exported until they reach a threshold concentration, after which they are recognized by sensor kinases that launch a phosphate group-transfer to a response regulator [138]. The Agr system, as described previously, is autoinduced through an extracellular ligand, namely the AIP, acting as a sensor of cell density and usually reaching its threshold around mid- to late-exponential phase [95, 139]. RNAIII, one of the produced effector transcripts of the Agr system, is described as a relevant bacterial virulence element in *S. aureus*, with some RNAIII-inhibiting peptide studies demonstrating its implication in biofilm formation and toxin production [140, 141]. In *S. shinii* IMDO-S216, the complete *agrBDCA* cluster was identified, while the *hld* gene was not found through manual annotation. The transcription of this gene and the activation of its promoter, P3, presumably lead to the expression of various genes involved in the synthesis of several virulence factors and exoproteins in *S. aureus* [94–96]. Even if virulence factors that pertain to *S. aureus*, such as δ-hemolysin, are usually not a concern for CNS strains [142–144], the quorum sensing mechanism is potentially implicated in bacterial communication mechanisms in *S. shinii* IMDO-S216.

In fermented meat products, elements pertaining to quorum sensing communication systems have been studied to decontaminate or control growth of *Listeria monocytogenes* without affecting beneficial bacteria in the process [145]. This highlights that quorum sensing systems may contribute to the quality and safety of fermented meat products. The presence of this genomic feature in nearly all *S. xylosus* strains, as well as in all strains from *S. nepalensis*, *S. pasteuri* JS7, and *S. equorum* KS1039, indicates the prevalence of a shared communication system (Fig. 1; Table S1). Also, as previously mentioned, the *hld* gene is present in twelve of the 38 strains selected for the comparative analysis. The identical sequence identity and query coverage percentages between *S. carnosus* and *S. condimenti* may be explained by their phylogenetic proximity [111]. Since April 2020, *S. carnosus* DSM 25,010 is considered as GRAS (Generally Recognized As Safe) [146] by the Food and Drug Administration, whereas this is not the case for the other bacteria referenced in this study. However, it has been shown that CNS isolates from blood culture usually express several virulence genes similar to those carried by *S. aureus* [147], which may not directly imply the pathogenicity of the bacterium.

## Conclusions

The genome of *Staphylococcus shinii* IMDO-S216 was found to contain a circular chromosome and eight plasmid replicons, that were then screened for the presence of genes coding for secondary metabolites. The complete gene cluster of lactococcin 972 was found on the main chromosome. In addition to this potential to generate a bacteriocin, other competitiveness factors were linked to several complete gene clusters located on the chromosome. These clusters coded for elements related to the D-alanylation of lipoteichoic acids on the cell wall; the synthesis of mevalonate and one of its by-products, namely staphyloxanthin; the synthesis of staphyloferrin A; and the Agr quorum sensing system. All these gene clusters were equally studied in 38 selected staphylococcal species with a nearly-complete or complete assembled genome, displaying an ubiquitous presence for the D-alanylation of lipoteichoic acids and the synthesis of mevalonate, and a more differentiated appearance for the other BGCs. Taken together, such factors may account for an enhanced adaptation and competitiveness of the studied strain in a meat fermentation environment, offering advantages like growth at low temperatures, intake of iron necessary for growth, efficient communication through a quorum sensing system, or protection against certain antimicrobial peptides. The combination of these competitiveness factors underlines the fitness of *S. shinii* IMDO-S216 in a fermented meat matrix. A further exploration of the expression of these gene clusters and the biosynthesis of their products will lead to an improved understanding of the functionality of this strain during meat fermentation.

### Electronic supplementary material

Below is the link to the electronic supplementary material.

Supplementary Material 1

## Data Availability

The complete assembled genome of *S. shinii* IMDO-S216 has been deposited in the European Nucleotide Archive at EMBL-EBI with BioProject accession number PRJEB56928 (http://www.ebi.ac.uk/ena/browser/view/PRJEB56928).

## Abbreviations

ABC: ATP-binding cassette
Agr: Accessory gene regulator
AIP: Autoinducing peptide
BGC: Biosynthetic gene cluster
Blastn: Basic local alignment search tool for nucleotides
Blastp: Basic local alignment search tool for proteins
CD: Coding sequence
CNS: Coagulase-negative staphylococci
FPP: Farnesyl diphosphate
GCC: Gram-positive, catalase-positive cocci
GRAS: Generally Recognized As Safe
GroP: Glycerolphosphate
HGT: Horizontal gene transfer
HMG-CoA: Hydroxy-3-methylglutaryl coenzyme A
IPP: Isopentenyl diphosphate
Lcn972: Lactococcin 972
LTA: Lipoteichoic acids
NRPS: Non-ribosomal peptide synthetase
ONT: Oxford Nanopore Technologies
RiPP: Ribosomally-synthesized and posttranslationally modified peptides
SA: Staphyloferrin A
